# Farmer’s climate smart livestock production adoption and determinant factors in Hidebu Abote District, Central Ethiopia

**DOI:** 10.1038/s41598-024-59967-8

**Published:** 2024-05-01

**Authors:** Desalegn Yayeh Ayal, Bassa Mamo

**Affiliations:** Center for Food Security Studies, College of Development Studies, Addis Ababa University, Addis Ababa, Ethiopia

**Keywords:** Adoption, Climate smart, Constraints, Compost, Extension, Livestock, Manure, Water, Biogas, Destocking, Climate sciences, Environmental sciences, Environmental social sciences

## Abstract

This study aimed to identify the status, determining factors, and challenges in adopting climate smart livestock production practices by farmers. Three-staged sampling techniques were used to select the research sites and 233 sample farmer household respondents. Data were collected mainly using a pre-tested structured questionnaire. Key informant interviews and focus group discussions were also conducted to complement the household survey data. Descriptive statistics and an ordered logistic regression model were applied to analyze the quantitative data. The result revealed that the most adopted practices were composting (85.41%) and manure management (70.39%) while the least adopted technologies were biogas generation (3.86%) and rotation grazing (22.32%). The adoption status of the sampled farmers was also categorized into low (19.74%), medium (67.81%), and high adopter (12.45%). The high cost of improved breed, use of manure for fuel, free grazing, lack of information and awareness were the major constraints to adopting the climate smart livestock production technologies. The result also revealed that education, grazing land, total livestock holding, and extension agent contact contributed significantly and positively to the adoption of smart livestock production technology, while the distance from the water source had an insignificant and negative effect on the adoption status of climate smart livestock production practices. The study suggests the relevance of the cooperation of stakeholders and strengthening extension services for the maximum benefits of climate smart livestock production.

## Introduction

The Food and Agricultural Organization of the United Nations^1^ estimates that agricultural production has to increase by 60% by 2050 to satisfy the expected demands for food and feed across the world. Most of the additional 2 billion people will live in developing countries^2^. Agriculture in Africa has a massive social and economic footprint. More than 60% of the population of Sub-Saharan African are smallholder farmers, and about 23% of SSA’s GDP come from agriculture. Yet Africa’s full agricultural potential remains untapped^3,4^. Agriculture is the backbone of Ethiopia’s economy^5,6^. At the national level, the sector accommodates 85% of employment and contributes to 90% of foreign exchange earnings. Most importantly 90% of the agricultural output is contributed by small-scale farming^7^. According to^8^ the agriculture sector contributed 39% of the GDP at the end of 2015.

Climate variability and extremes are a real danger to the sustainable development of the agriculture sector^9,12^. The adverse impacts of climate variability and extremes are manifested in Ethiopia through drought, flood, increase in temperature, and change in rainfall distribution which have direct and indirect effects on livestock production^10–13^. In reverse, the business-as-usual livestock production system contributes both directly and indirectly to climate change through the release of greenhouse gases (GHG) emissions in the form of carbon dioxide (CO2), methane (CH4), and nitrous oxide (N2O)^14,15^. Climate-Smart Agriculture (CSA) is a proven solution to address the causes and impacts of climate change and ensure food security^9,16,21^. Cognizant the adverse effect of climate variability and extremes; Ethiopia adopted climate change adaptation and mitigation policy framework called Climate Resilient Green Economy (CRGE) and Nationally Determined Contributions to mainstream the environmental issues to all development efforts in which the Climate Smart Agriculture (CSA) development is the main component^17,49^.

In the context of the CSA approach, livestock production is an agricultural sub-sector that is targeted for climate change adaptation and mitigation, especially in arid and marginal lands characterized by the mobility of live animals, less gestation period, and less water requirement^18,19^. In the effort to support local livestock raising farmers in the Hidebu Abote district local government and partner organizations have implemented different development programs out of which the Growth and Transformation Plan (GTP I and II1), Agricultural Growth Program (AGP), Sustainable Land Management (SLM) and ten-year growth and transformation plan to integrated a climate smart livestock production since the year 2010/2011. In AGP-II climate smart livestock production practices like zero-grazing, cut and carry system, area closure, fattening, breed improvement for dairy livestock production, Artificial Insemination (AI), animal health services, and composting are included in the document and implemented since 2015^8,20^. However, climate smart technologies are biophysical and socioeconomic specific, thus, what is smart in one locations and a given community may not be smart in other location^16,21^. Therefore, understanding climate smart livestock production practices and adoption status as well as determinate factors at local level is utmost important.

There have been insufficient empirical studies and scientific evaluations of context-based climate smart livestock production programs in the study area and elsewhere in Ethiopia so far to identify the adoption status, determining factors, and challenges in using the practices^16^. To address that gap this study aimed to identify the status of adoption, analyze determining factors and challenges in adopting climate smart livestock production practices, and forward problem-oriented context specific strategic action to enhance adoption and scaling up the practice by farmers in the study area and other parts of Ethiopia. Therefore, this paper could shed light on the micro level literature on climate smart livestock production determinants and serve as a baseline for future research.

## Literature review

The livestock sector could contribute to poverty reduction, food security, and agricultural development^22^. It contributes 40% of the global agricultural gross domestic product (GDP), means of livelihood, and nutrition security for 1.3 billion people in the globe^22–24^. Likewise in Ethiopia, livestock production contributes 19% of the national GDP, 16–19% of the total export earnings, and 30% of agricultural employment^25^. Besides, it has sociocultural values, and plays a crucial role in soil fertility management, promoting saving, draught power for crop cultivation and transporting goods and people, and fuel for domestic cooking^26^.

Even though, Ethiopia ranked first in Africa and fifth in the world in livestock population, the sector is characterized by traditional farming system and low production & productivity^27^. The livestock sector performance is compromised by interrelated factors such as population pressure induced land degradation, biodiversity loss, deforestation as well as lack of technology, uninformed policy and poor political commitment. These biophysical and institutional factors exacerbated the adverse impact of climate variability and extremes^28^. The cumulative impacts of climate variability and extremes push the poor farmers and pastoralists under poverty and worsen their food insecurity^9,12,22,28^. Ethiopian livestock sector is experiencing a reduction of production and productivity. Whereas, global and national consumption demand from the livestock sector is expected to remarkably increase to feed the fast population growth. For instance, Shapiro, et al.^29^ reported that the annual consumption of beef projected to grow by 200%, mutton and goat meat by 114.3% each, camel meat by 83.3%, chicken meat by 80%, and eggs by 355.6%.

## Materials and methods

### Description of the study area

**Figure 1:**
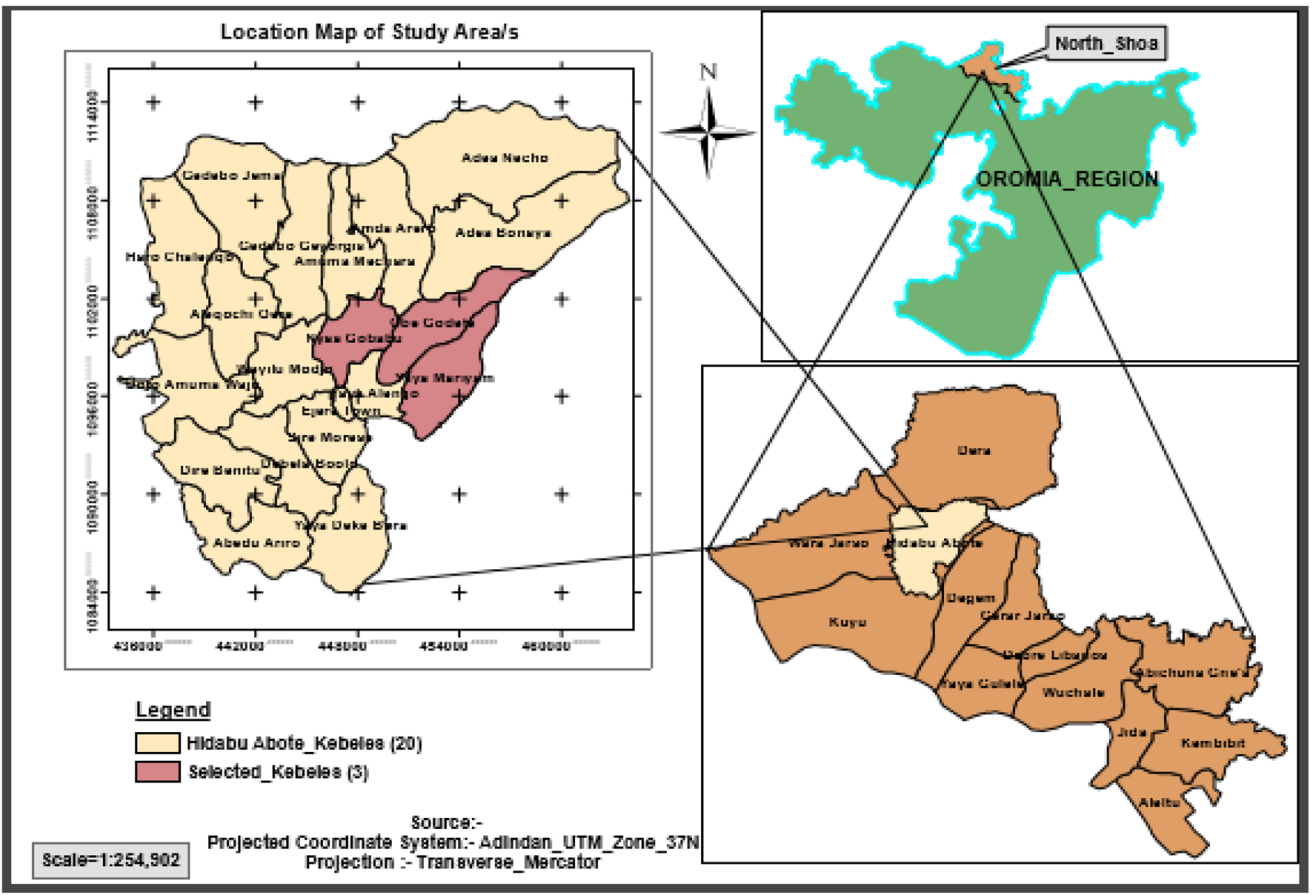
Map of the study area (Source: Own contraction using *GIS, 2020*).

Hidabu Abote district is located between 9° 47′ 15″ and 10° 0′ 45″ N and 38° 26′ 15″–38° 38′ 45″ E astronomically, an altitude ranging from 1160 to 3000 m above sea level (Fig. 1). It has a total population of 104, 442 of which 15, 086 are agriculturally based households. The total area of the district is 50,870.39 ha of which 32, 917 (64.7%) ha is used for agricultural production. Agriculture contributes much to meeting the major objectives of farmers such as food supplies and cash needs. It is characterized as rain-fed and subsistence nature with traditional farming system. The agricultural system is a mixed farming type where crop and livestock production are practiced jointly. The dominant crops grown in the study district are *teff*, sorghum, wheat, chickpea, faba bean, and lentils. The number of livestock resources in the study area were; cattle (81,156), sheep (23,899), goats (47,596), horses (439), donkeys (12,528), mules (173), poultry (43,814) and honeybee colonies (15,648).

### Research design

An explanatory mixed research design was applied in this research. Explanatory mixed research sets out to describe and interpret the questions and looks at the study units to explain, compare, contrast, classify, analyze, and interpret the entities, and the events that constitute the study^30^. Different socio-economic, institutional, and demographic situations were described and explained adequately. Household survey, key informant interview (KII), Focus Group Discussion, and field observation as methods enabled the researcher to explore and explain the phenomenon and establish arguments.

The study was performed in accordance with the College of Development Studies, Addis Ababa University research ethics guidelines. Accordingly, the present study was approved by the College of Development Studies’ ethical review board. Informed consent was obtained from all respondents. Secondary data were also collected from relevant sources such as articles, proceedings, journals, scientific reports, MoA, CSA, Zonal, and district annual reports which were vital to the study.

### Sampling technique and sample size determination

This study followed a multi-staged sampling technique, where a combination of sampling techniques were used to select the study sites and participants. A purposive sampling method was employed to select the study district and the three *Kebeles* namely Yayamarami, Kobigodeti, and Gneagebabu due to their livestock production potential and climate change related risks. The study sites were also affected by population pressure. The livestock producing farmers were selected using a simple random sampling method. This approach was employed due to its ability to provide a fair and unbiased representation of livestock producer farmers with different socioeconomic and demographic characteristics. This sampling technique ensured that every member of the population had an equal opportunity for selection, thereby enabling the findings to apply to the entire population sharing a similar context. A simple random sampling method could enhance the reliability and applicability of the study's results to a broader spectrum of livestock-producing farmers in similar contexts.

The key informants from livestock development agents, kebele managers, knowledgeable community members (five in each kebeles) and heterogenous focus group discussion participants from youth, female-headed & male-headed households, knowledgeable community members (six up to seven members in each kebeles) were also selected purposively due to their skill and knowledge to explain the status and determinants related to climate smart livestock production. Reference^31^ provides a simplified formula to calculate sample sizes. This formula was used to calculate the sample size as shown below in Table 1.

$${\text{n}}=\frac{N}{1+N{(e)}^{2}}=\frac{1447}{1+1447{(0.06)}^{2}}\approx 233$$

where n is the sample size, N is the population size (1447), and e is the level of precision (6%).

**Table 1:** Sampled households in selected *kebeles.*

Name of sampled *kebeles*	Total household	Sampled household	% from sample size
Yayamarami	540	87	37.34
Kobigodeti	462	74	31.76
Gneagebabu	445	72	30.90
Total	1447	233	100

Source: HALFRDO (2020).

### Data analysis method

To address the objectives of this study, a mix of data analysis methods were employed. Qualitative data were analyzed using thematic content analysis. Descriptive statistics such as mean, minimum, maximum, percentages, frequencies, and standard deviation were applied to describe the demographic, socioeconomic, farm characteristics, and institutional characteristics of the study area. An ordered logit regression model was used to analyze socio-economic determinants of the farmers’ adoption status of climate smart livestock production practices. The ordered logit regression model was used because our dependent variable (farmer’s adoption level) is measured in ordinal scale ranging from ‘low adoption’ to ‘high-adoption’ rate^32^. Inferential statistics like the one-way ANOVA-F test and Chi-square were used to compare the difference among adoption categories for different continuous and categorical variables and also whether the difference is significant or not.

According to Ref.^32^, the ordered logit regression model is expressed as:

$${\text{P}}\left({\text{Yi}}>j\right)=g\left(X\beta j\right)=+\frac{{\text{exp}}(\alpha j+Xi\beta j)}{1+\{{\text{exp}}\left(\alpha j+Xi\beta j\right)\}}, j=1.2,..,M-1$$

where M is the number of categories of the ordinal regressed. From the equation stated above, the probabilities that Y will take on each of the values 1, … M are equal to:

$$P\left(Yi=1\right)=1-g(Xi\beta j)$$

$$P\left(Yi=j\right)=g\left(Xi\beta j\right), J=2,\dots ,M-1P\left(Yi=M\right)=g\left(Xi\beta M-1\right)$$

$$Yi={\beta }_{0}+{\beta }_{1 }{X}_{1}+{\beta }_{2}{X}_{2}+{\beta }_{3}{X}_{3}+\cdots {\beta }_{n}{X}_{n}+\mu i.$$

The dependent variable Yi = level of usage of climate-smart livestock production practices (high user = 3; medium user = 2; low user = 1). X1… X represents the explanatory variables; ${\beta }_{1}$…${\beta }_{n}$ represents the parameters of the explanatory variables; and ${\beta }_{0}$ represents the intercept, while $\mu i$ represents the error term.

### Variable description

#### Dependent variable

The adoption quotient, developed by^33^ is the dependent variable used in this study. The adoption quotient for an individual farmer was calculated based on the adoption scores gained by the farmer for the adoption of climate smart livestock production practices. A total of 8 climate smart livestock production practices (improved breed, composting, manure management, fodder planting, feed conservation, rotational grazing, biogas generation, and destocking) were used for the calculation of the adoption quotient.

$${\text{Adoption Quotient }} = \, \left( {{\text{Total adoption scores gained by farmer}}/{\text{Maximum adoption score}}} \right) \times {1}00.$$

Depending on the adoption quotient, sampled households were divided into three categories for analysis such as low adoption ≤ (Mean − SD), medium adoption = (Mean ± SD), and high adoption ≥ (Mean + SD); and also, the same three categories applied for ordered logistic regression analysis^34^.

#### Independent variables

The independent variables that affect the farmers’ adoption of climate smart livestock production practices are the combined effects of various factors such as household demographic characteristics, socio-economic, and institutional factors in which farmers operate^16,21,35^. Based on the review of related literature and a total of 13 potential explanatory variables were considered in this study and examined for their effect on the adoption of climate smart livestock production practices by farmers practices (Table 2).

**Table 2:** Summary of variable definition.

Types of variables	Definition	Types variables	Measurement	Expected sign
Sex	Sex of the sampled households	Dummy	1 for male, 0 female	+
Education	Education level of sampled households	continuous	School years	+
Family size	Family size of sampled households	Continuous	AE	+
Livestock income	Livestock income earned from livestock	Continuous	Birr	+
Saving and Credit use	Saving and Credit use of the sampled households	Dummy	1 for saving and credit use and 0 for not use	+
Extension contact	Extension service of the sampled households	Dummy	1 for extension contact, 0 for not contact	+
Information on climate change	Information on climate change by sampled households	Dummy	1 have information, 0 not have information	+
Landholding	Total number of land that sampled households have	Continuous	ha	+
Livestock holding	Total livestock owned by sampled households	Continuous	TLU	+
Grazing land	Total grazing hold by sampled households	Continuous	ha	+
Distance from a water source	Distance from the nearest water source	Continuous	Walking minute	−
Experience	Experience of sampled households on livestock production practices	Continuous	Years	+

After coding and feeding the collected primary data into the computer, STATA version 15 was used for analysis.

## Results and discussion

### Socio-economic and demographic characteristics of respondents and implications to climate smart livestock production

Table 3 shows 26.18% of the sample households were female headed and the remaining 73.82% were male headed households. The result of the Chi-square shows the existence of insignificant relationship between sex with climate smart livestock production adoption level (X^2^ = 1.229, P = 0.541). However, the majority of male headed sampled households (69.19%) were in a medium level adoption status as compared with female headed households (63.93%). Likewise, female headed households fall under low level climate smart livestock production found to be more than male headed household respondents in the same category. About 40%, 72.63% and 84.06% sample household low category adopters, medium level adopter and high level adaptors have access to saving and credit services respectively. However, the Chi-square test result shows the statistically insignificant relationship between the saving and credit access to smart livestock production technology adoption status (X^2^ = 15.426, P = 0.144). A majority of high and medium level category climate smart livestock production technology adopters have saving and credit services means; saving and credit services positively influencing the adoption of climate smart livestock production technology.

**Table 3:** Descriptive Statistics for dummy variables (%).

Variables	Description	Low	Medium	High	Total	X^2^	Sig
		%	%	%	%		
Sex	Female	24.59	63.93	11.48	26.18		
	Male	18.02	69.19	12.76	73.82	1.229	0.541
Saving and Credit service	Yes	40	72.63	84.06		3.878	0.144
	No	60	27.37	15.94			
Extension service	Yes	26.59	69.67	100		15.426	0.00
	No	73.41	30.33	0			
Climate information	Yes	14.77	72.72	93.49		11.566	0.003
	No	85.23	27.28	6.51			

Table 3 shows all high level category adaptors and majority (69.67%) level category adaptors have access to extension services. Whereas 73.41% of low level climate smart livestock production have no access to extension service. There is a significant statistical difference between farmers’ choice of climate smart livestock production practice about the extension service (X^2^ = 5.038, P = 0.000). This shows that extension service is a basic determining factor for the adoption of climate smart livestock production technology. This confirms access to extension services favors to use more technology as compared with none extension users. Likewise, the majority of high level category adaptors (93.49%) and majority (73.72%) level category adaptors have access to climate information. Farmers access to climate information influence significantly the adoption of climate smart livestock production technology (see Table 3). Key informants’ and FGD participants also underlined that initial capital, access to information and extension services motivate farmers to adopt climate smart livestock production technology. It seems access to extension positively influences farmers to adopt climate smart livestock production technology.

The age of respondents ranges from 23 to 77 years. Table 4 shows that the average age of the respondents were found to be 44.37, 45.96, and 47.41 for low, medium, and high adoption level categories respectively. The one-way ANOVA result indicates that there is a significant difference between the groups of adoption categories (F = 2.011, P = 0.001). The result asserts the older age stimulate climate smart livestock production technology adoption and hence, it is an important factor in enhancing the adoption of climate smart livestock production technologies.

**Table 4:** Summary of socioeconomic and demographic characteristics.

Variable	Measurement	Mean	Std. dev	Min	Max
Age	Year	45.82	11.08	23	77
Education	School years	3.63	3.91	0	13
Family size	AE	5.42	2.06	1.6	12.15
Landholding	ha	2.6	0.99	0.25	5.4
Grazing land	ha	0.46	0.35	0	2
Livestock holding	TLU	5.77	2.57	0.84	13.15
Livestock income	Birr	11,300.48	13,926.64	0	66,000
Distance from water	Walking minute	13.70	10.85	2	60
Experience	Years	12.39	5.78	2	52

The average years of schooling in the study area was 3.63, with a minimum of zero years (illiterate) and a maximum of 13 years of schooling. The mean education level for the household head that falls in the adoption categories of low, medium, and high were 2.61, 3.64, and 5.21 (see Table 4). Table 5 shows the existence of an educational level significant difference between the groups of adoption categories (F = 1.589, P = 0.040). Relatively higher the educational level favors the adoption of climate smart livestock production technologies. The possible reason is that a higher level of education could increase the farmers access to the extension and credit services, take informed risks, receive relevant climatic information, understand, and adopt new technology. KII and FGD participants reported that educated farmers mostly participate in various local level committees, administration and trainings. Therefore, it seems certain that the educated farmers could adoption climate smart livestock production to respond to the adverse impact of climate variability and extremes and ensure food security sustainably.

**Table 5:** Climate smart livestock production practices adoption status to continuous explanatory variables.

Variables	Adoption	Mean	Std. Dev	F-value	P-value
Age	Low	44.37	11.27	2.011	0.001
	Medium	45.96	10.86		
	High	47.41	12.08		
Education	Low	2.61	3.01	1.589	0.040
	Medium	3.64	3.94		
	High	5.21	4.57		
Family size	Low	4.93	2.06	1.469	0.021
	Medium	5.44	1.94		
	High	6.08	2.54		
Landholding	Low	1.88	1.03	1.327	0.047
	Medium	2.03	0.87		
	High	2.52	1.25		
Grazing land	Low	0.29	0.26	2.669	0.000
	Medium	0.46	0.33		
	High	0.69	0.45		
Livestock holding	Low	4.711	2.37	2.361	0.000
	Medium	5.84	2.62		
	High	7.14	1.89		
Distance from water	Low	18.46	12.96	1.218	0.226
	Medium	12.70	10.24		
	High	11.55	8.33		
Experience	Low	12.19	4.89	2.056	0.004
	Medium	14.10	5.26		
	High	17.24	8.91		
Livestock income	Low	255.6	441.7	1.477	0.019
	Medium	532.4	309.9		
	High	675.2	429.1		

Table 5 shows that the average family size of the sample household was 5.42, with a minimum of 1.6 and a maximum family size of 12.15 in terms of adult equivalent (AE). The average family size of the sample farmers shows the potential labor endowment in the study area. The average landholding size of the sample farmers was 2.6 hectares with a minimum of 0.25 hectares and a maximum of 5.4 hectares. The mean landholding for the sample farmers falls in the adoption categories of low, medium, and high were 1.88, 2.03, and 2.52 with a standard deviation of 1.03, 0.87, and 1.25 respectively. The result shows there is a significant relationship between climate smart livestock production technologies adoption in all categories (F = 1.327, P = 0.047). This shows that landholding size is a basic determining factor for the adoption of climate smart livestock production technologies.

The average total own grazing land of the sample farmers was about 0.46 hectares with a range of 0 hectares to 2 hectares. The mean grazing land for the sample farmers fall in the adoption categories of low, medium, and high were 0.29, 0.46, and 0.69 with a standard deviation of 0.26, 0.33, and 0.45 respectively. Table 5 shows that there is a significant difference between climate smart livestock production technology adoption categories (F = 2.669, P = 0.000) and landholding in the study area.

On average, the livestock holding was 5.77 per sample farmer with a minimum of 0.84 and a maximum of 13.15 in TLU. The mean livestock holding of the sample households in the Tropical Livestock Unit (TLU) were 4.71, 5.84, and 7.14 for low, medium, and high adoption categories with a standard deviation of 2.37, 2.62, and 1.89 respectively. The result shows there is a significant (F = 2.361, P = 0.000) relationship between climate smart livestock production technology adoption and livestock holding. This indicates that farmers who have more livestock could participate in climate smart livestock production practices.

The average yearly livestock income of the sample farmers was Birr 289.8 US dollars with a standard deviation of 13,926.64. The maximum livestock income was 1697.3 US dollars with a minimum of zero US dollars. The result indicates that farmers could generate more income from livestock production in the study area. Table 5 shows that the mean livestock income for the sample farmers falls in the adoption categories of low, medium, and high were 255.8, 532.4, and 675.2 US dollars respectively. Similarly, the standard deviation of livestock income for low, medium, and high adoption categories were 441.8, 309.9, and 429.1.72 respectively. Table 5 shows that income from livestock brought a significant difference between the groups of adoption categories (F = 1.477, P = 0.019). This result explains low category climate smart livestock production technology adaptors income from the livestock sector is more than high and medium level climate smart livestock production adaptors. This could be due to the fact that the study sites are dominated by crop farming and hence, high and medium category climate smart livestock technology adaptors use compost, manure, biogas and rotation grazing to manage the cultivated land fertility. Then the economic benefit of climate smart technology in the study area could be explain indirectly through crop productivity, chemical fertilizer cost, and land management.

The mean distance from home to the nearest water source was 13.7 walking minutes with a minimum of 5 min and a maximum of one hour. The average distance from home to the nearest water source of the sample farmers in a walking minute were 18.46, 12.70, and 11.55 for low, medium, and high adoption categories with a standard deviation of 12.96, 10.24, and 8.33 respectively. The result shows that there is insignificant (F = 1.218, P = 0.22) relationship between climate smart livestock production technology adoption categories and water point distance.

The average years of farming experience in the study area was 12.39 years, with a maximum of 52 and a minimum of 2 years. The average experience of the sample farmers falls in the adoption categories of low, medium, and high were 12.19, 14.10, and 17.24 with a standard deviation of 4.89, 5.26, and 8.91 respectively. The result of one-way ANOVA shows that there is a positive significant (F = 2.056, P = 0.004) difference between climate smart livestock production technology adoption categories and farming experience in the study area. This is because as one becomes skillful in the methods of livestock production, he/she will be better at adopting climate smart livestock production practices.

### Adoption of climate smart livestock production status

The eight climate smart livestock production practices that have been promoted by development actors and adopted by farmers at different levels were rotation grazing, improved fodder, destocking, feed conservation, composting, manure management, genetic breed improvement, and bio-gas generation. Table 6 shows that the widely adopted practices were composting (85.41%) and manure management (70.39%) while the less adopted practices were bio-gas generation (3.86%). Adoption of destocking (63.95%), improved breed (60.09%), fodder (29.18%), and rotation grazing (22.32%) were intermediary adopted climate smart livestock production practices. Table 6 depicts that male headed households were found to be more climate smart livestock production practices adopters in all practices. It seems male headed households were in a better position to practice diverse climate change adaptation and mitigation strategies than the female-headed. As reported by ^36^ this could be due to the fact that male headed households are more likely to have access to technologies and climate change and variability information than female-headed households.

**Table 6:** Types of climate smart livestock production technologies practiced in the study area.

Climate smart practices	Adopters				Non adaptors			
	Female	Male	Total		Female	Male	Total	
	%	%	Fre	%	%	%	Fre	%
Improved breed	55.74	61.63	140	60.09	44.26	38.37	93	39.91
Manure management	60.66	73.84	164	70.39	39.34	26.16	69	29.61
Fodder planting	32.79	27.91	68	29.18	67.21	72.09	165	70.82
Feed conservation	55.74	70.35	155	66.52	44.26	29.65	78	33.48
Composting	77.05	88.37	199	85.41	22.95	11.63	34	14.59
Rotational grazing	19.67	23.26	52	22.32	80.33	76.74	181	77.68
Biogas generation	8.2	2.33	9	3.86	91.8	97.67	224	96.14
Destocking	60.66	65.12	149	63.95	39.34	34.88	84	36.05

In the study area sample farmers were grouped based on the adoption quotient derived from calculated adoption scores and compared the result with the arithmetic of adoption mean and standard deviation that is mentioned in Table 7. The result of the analysis of the adoption quotient of farmers indicates that the minimum adopted practices were 0% and the maximum adoption practice was 87.5%. The mean adoption quotient is 0.4796 and the standard deviation is 19.96 (see Table 7) which shows that there are encouraging adoption practices that need to be scaled up in the study area.

**Table 7:** Summary of adoption quotient of sampled households (in percentage).

Variable	Observation	Mean	Std.Dev	Min	Max
Adoption quotient	233	47.96	19.96	0	87.50

Source: Own survey (2020).

Table 8 shows that 19.74%, 567.81%, and 12.45% of the sample farmers were categorized under low, medium, and high adoption levels respectively for Chi-square and ordered logistic regression models. This indicates that the majority of the respondents were categorized under medium adoption level while a few of them fall at low and high climate smart livestock production technology adopter’s category.

**Table 8:** Adoption categories of sampled households.

Adoption status	Frequency	Percentage
Low	46	19.74
Medium	158	67.81
High	26	12.45
Total	233	100

Source: Own survey (2020).

### Determinate factors of climate smart livestock production

#### Diagnostic test

An ordered logit regression model was applied to estimate the determinants of farmers ‘choices of adopting climate smart livestock production practices that aimed to reduce the adverse impact of climate variability and extremes. The dependent variable of the model is the category of users of climate smart livestock production practices (i.e., high, medium, and low). Before running the ordered logistic regression model, different econometric assumptions were tested using appropriate techniques. The existence of multicollinearity between the explanatory variables was checked by using VIF for the continuous variable while the coefficient of contingency was used for the dummy variable. As a rule of thumb VIF values, less than 10 are said to be a weak association among explanatory variables.

Therefore, in this study, the computational results of the VIF for continuous variables are lower than 1.58 and the mean of VIF is 1.26 which confirmed the non-existence of multicollinearity problem among the continuous predictor variables and was included in the model. Besides, the values of the contingency coefficient regarding dummy variables were less than 0.29 which is less than the rule of thumb of 0.75 implying a weak degree of association among the variables considered. Moreover, from Table 9 Prob > chi^2^ = 0, which indicates the fitness of the model during analysis.

**Table 9:** Result of ordered logistic regression model.

Adoption status	Coef	Std. Err	Z	P > z
Sex	0.174	0.354	− 0.49	0.623
Education	0.118***	0.040	2.94	0.003
Family size	0.058	0.081	0.71	0.475
Landholding	0.166	0.192	− 0.87	0.386
Grazing land	1.470***	0.499	2.94	0.003
Total livestock holding	0.128*	0.069	1.85	0.050
Livestock income	0.000	0.000	0.57	0.567
Credit use	0.079	0.310	− 0.25	0.802
Extension contacts	1.396***	0.529	2.64	0.008
Distance from water source	− 0.043***	0.014	− 3	0.003
Climate information	0.112	0.366	0.31	0.76
Experience	0.039	0.027	1.44	0.15
cut1	1.233	0.737		
cut2	5.360	0.837		

*** and * Significant at 1% and 10% level respectively.

Source: Model result (2020).

Hence, the parameter estimates of the ordered logit model were used to provide the direction of the effect of the independent variables on the dependent (response) variable, whereas estimates represent neither the actual magnitude of change nor the probabilities. The marginal effects of marginal probabilities are a function of probabilities and measures expected to change within the probabilities. In the subsequent section, only the variables that were statistically significant at less than or equal to 10% probability levels are interpreted and discussed.

**Education:** The result of the ordered logit regression model showed that educational status is statistically significant and has a positive influence on the level of adoption of climate smart livestock production technologies (P = 0.003). It indicates that educated sampled farmers are more likely to be in the higher category. The marginal effect in Table 10 shows that as education increases by one school year keeping other variables constant, the probability to be in the lower adoption category is likely to decrease by 1.5%, the probability of being in the medium category likely increases by 0.6%, and the probability to be in the higher category likely increase by 0.9%. This confirms r that the more farmers invest in education, the more they gain relevant skills and knowledge about nature-based farming. Education could also expose them to various types of technologies and information. The result also pointed out that better exposure to education increases farmers’ understanding of the benefits and constraints of climate smart livestock production technologies. A positive impact of education on technology acquisition is generally expected as it enhances farmers’ ability to acquire and analyze new ideas, and provides specific or general skills that contribute to livestock productivity. References^37,38^ also reported that education gives farmers the ability to perceive, interpret, and respond to new information much faster than farmers with lower education levels (non-educated). Thus, those household heads with better education levels have a higher probability of adopting best practices.

**Table 10:** Marginal effects after ordered logit model.

Adoption status	Marginal effect	Marginal effect	Marginal effect
	(dy/dx) for low adopter	(dy/dx) for medium adopter	(dy/dx) for high adopter
Sex	0.021	− 0.008	− 0.014
Education	− 0.015	0.006	0.003
Family size	− 0.007	0.003	0.004
Landholding	0.021	− 0.008	− 0.013
Grazing land	− 0.187	0.073	0.003
Total livestock holding	− 0.016	0.006	0.010
Livestock income	0.000	0.000	0.000
Saving and credit use	0.010	− 0.004	− 0.006
Extension contacts	− 0.250	0.180	0.005
Distance from water source	0.005	− 0.002	− 0.003
Climate information	− 0.014	0.006	0.008
Experience	− 0.005	0.002	0.003

Source: Model result (2020).

**Grazing Land: **Table 10 shows grazing land has a positive statistically significant (P = 0.003) influence on the level of adoption of climate smart livestock production practices. The result indicates that farmers who have larger grazing land are more likely to be in the higher category of climate smart livestock production adoption in the study area. The marginal effect shows that as grazing land increases by one hectare, assuming other factors are constant, the probability of being in a lower category decrease by 18.7%, the probability of being in the medium category increases by 7.3%, and the probability to be in the higher category increase by 11.3%. This is because relatively large size grazing land is vital to adopting climate smart livestock production technologies such as growing improved fodder, feed conservation (hay production), etc. which require more space.

**Total livestock holding:** As expected, total livestock holding was found to be significantly positively (P = 0.050) associated with the adoption status of climate smart livestock production technology. Table 10 shows that as livestock increased by one unit, keeping other variables constant, the probability to be in the lower category decreased by 1.6%. Whereas, the probability to be in the medium and higher category increased by 0.6% and 1% respectively. Thus, possessing more livestock encourages farmers to adopt climate smart livestock production practices. This could be attributable to the fact that livestock is a liquid asset and serves as a startup’s initial capital to implement climate smart production technology. Besides, livestock possession in rural Ethiopia in, general, and in the study area, in particular, is considered an indicator of income level and hence wealth status of the households. On the other hand, some of the livestock types, such as donkeys and horses, are still important means of transport for goods and human beings in the study area. The result affirms similar claims made by previous researchers such as^38,40^ who reported livestock holding motivates the adoption of CSA related practices.

**Extension contacts:** Table 10 shows that farmers’ frequent contact with development agent has a statistically significant positive effect (P = 0.005) on climate smart livestock production technology adoption. Therefore, farmers who have frequent contact with the extension agent are more likely to be in the higher category and less likely in the lower category to adopt climate livestock production in the study area (see Table 10). The possible explanation the development agent could share relevant information and skills about climate smart livestock production on one hand and influence the perceptions of farmers about the exposure and sensitivity of the livestock sector to climate variability and extremes. Farmers with more access to information and technical assistance on agricultural activities have more awareness about the consequences of climate change. Based upon the innovation diffusion theory, farmers who have contact with the extension agents’ access to vices and facilities tend to be more progressive and receptive to new technologies. Agricultural extension services could play a crucial role identifying and sharing environmentally feasible and affordable climate change adaptation and mitigation measures. Besides, the extension agents frequently visit and follow up could help to establish smooth relationships and build trust with farmers. This result agrees with the finding of^41,42^ who confirmed that contact with extension agents increases the likelihood of the adoption of climate smart agricultural practices. Moreover, this finding is consistent with those of^37,38^ who analyzed the adoption of soil and water conservation techniques and composting in different parts of Ethiopia respectively. Also, Refs.^29,39,43^ reported similar findings on composting technology adoption in China, Kenya, and Burkina Faso respectively.

**Distance from water source:** water access to demotic use could affect the of climate smart livestock prodcution adoption status significantly negatively (P = 0.003) (see Table 9). The more distance between the water source and the residence, the lesser the will be to adopt climate smart agriculture livestock production. The marginal effect shows (Table 10) a unit increase (1 walking minute) from the resident to the nearest water source the probability of the farmers of being in the lower category would increase by 0.5%, the probability of the farmers being in a medium category would be decreased by 0.2% and the probability being in the higher category would be decreased by 0.3% in the study area (Table 8). This could be attributed to the fact that the more distant the water source from the residential area, the greater would be the cost of consuming time and labor, adding a burden for livestock management and supervision. Moreover, thefarmer whose water source is far from their residence is less likely to continuously water his/her livestock as compared to those whose water source is nearer to their home. Thus, it is expected that farmers who live near the water source are likely to have regular watering of their livestock, hence motivated to respond to climate change in their agricultural activities. The result is similar to the finding of ^44^.

$$\begin{gathered} {\text{Number of obs}} = {233} \hfill \\ {\text{LR chi2}} \left( {{12}} \right) = { 57}.{56} \hfill \\ {\text{Prob}} > {\text{ chi2}} = 0 \hfill \\ {\text{Pseudo R2}} = 0.{1465} \hfill \\ {\text{Log likelihood }} = - {167}.{65183} \hfill \\ \end{gathered}$$

Table 11 shows that the mean predicted values of low, medium, and high adoption categories were 0.197 (19.7%), 0.68 (68%), and 0.133 (13.3%) which is almost similar to the mean value. This indicates that the model correctly predicted the value.

**Table 11:** Predicted probability for adoption category.

Variable	Obs	Mean	Std. Dev	Min	Max
Predicted probability (low)	233	0.197	0.168	0.004	0.829
Predicted probability (medium)	233	0.680	0.127	0.167	0.775
Predicted probability (High)	233	0.123	0.120	0.003	0.794

Source: Model result (2020).

### Constraints to climate smart livestock production adoption

Climate smart agricultural technology adopter farmers are facing multiple reinforcing challenges in the study area. The major challenges that prohibited these farmers from adopting climate smart livestock production technologies were identified and presented in Table 12. KII and FGD participants underlined that the high cost of improved breed, lack of feed availability, use of manure for fuel and crop residue for animal feeding, lack of free grazing, low awareness, small grazing land size and lack of finance were the major challenges to adopting climate smart livestock production technology in the study area. This finding is in agreement with the study of^2^ that described the number of challenges faced by climate smart agriculture in relation to the conceptual understanding, practice, policy environment, and financing of the approach. Proper pasture management through rotational grazing would be the most cost-effective way to mitigate greenhouse gas emissions from feed crop production and through grassland carbon sequestration. Animal grazing on pasture also helps reduce emissions attributable to animal manure storage^19,45^.

**Table 12:** The main constraints in adopting climate smart livestock production in the study area.

Types of climate smart livestock production practice	Major constraints	Count	%
Main reason not to use improved breed	High cost of improved breed	36	38.71
	Lack of technical practice	14	15.05
	Low awareness of improved breed	11	11.83
	Low availability of feed	20	21.51
	Far distance from the health service	8	8.60
	Lack of interest	4	4.30
	Other	0	0
Main problems to prepare compost	Use manure for fuel and crop residue for animal feeding	16	47.06
	Shortage of compost materials	8	23.53
	Lack of labor	6	17.65
	Un availability of water	3	8.82
	Low awareness	1	2.94
	Other	0	0
Reasons not to use fodder planting	Problem of free grazing	96	58.19
	Land allocation for crop production	27	16.36
	Small land	13	7.88
	Unavailability of water through a year	21	12.73
	Unavailability of improved fodder seed	7	4.24
	Other	1	0.60
Main problem of not use feed conservation	Limited of feed storage	53	67.95
	Low awareness	25	32.05
Reasons not to use rotational grazing	lack of awareness and knowledge on benefit	43	23.76
	Small grazing land	97	53.59
	Weak land use policy enforcement	21	11.60
	communal ownership of grazing land	20	11.05
	Other	0	0
Reasons not to practice biogas	Lack of information and awareness	102	45.54
	Lack of finance	98	43.75
	Small quantity of manure	20	8.93
	Other	4	1.79
Reasons not to use destocking	Low awareness on destocking	58	69.05
	Cultural attitude	21	25.00
	Other	5	5.95

Source: Own survey (2020).

Non-adopting farmers reported different primary constraints as a reason for not practicing improved breeds. Key informants indicated that improved livestock breeding practice has the potential to address the three pillars of climate smart livestock production (productivity and income, adaptation, and mitigation) and has widely promoted in the study area by government and non-governmental organizations for a long period. Table 12 shows that 38.71% of sample farmers were unable to adopt the improved breed mainly due to the high cost of the improved breed, lack of technical practice 15.05%, low awareness 11.83%, low availability of feeding 21.51%, distance from the health service 8.6%, and lack of interest 4.3%.

Composting is one of the climate smart livestock production practices widely promoted by the regular extension government programs, sustainable land management, and Agricultural Growth Programs (AGP). Composting is an environmentally friendly and cost effective soil fertility management technique. It could help to reduce the greenhouse gas concentration through methane reduction, offset nitrous oxide (N2O) released by the application of inorganic fertilizer, and stabilize the soil moisture and organic matter content^1^. As indicated in Table 12 about 14.59% of non-adaptors were not using compost. The main reasons were the use of manure for fuel and crop residue for animal feeding (47.06%), shortage of compost materials (23.53%), lack of labor (17.65%), unavailability of water (8.82%), and low awareness (2.94%). Compost making is a dominantly adopted practice (85.41%) by sample farmers in the study area. The result concurs with previous findings by^13^ who identified that family labor and poor awareness are major constraints to adopting composting.

Proper conservation feeding to livestock production is an efficient and productive feeding practice that enhances the yield and compensates for the shortage of feed occurring during dry seasons which helps to adapt to climate change impacts. It also contributes to a high animal feed conversion rate and reduces the amount of methane (CH4) gas released per head of animals to mitigate greenhouse gas release to the ambient atmosphere^19^. Table 12 depicts 33.48 of sample farmers were not able to adopt the practice mainly due to limited feed storage (67.95%) and low awareness (32.05%).

Fodder is planting trees together with crops on the farm. These are trees that produce or are primarily used for fodder (for animal feed), or fuelwood production or that provide other benefits such as reducing runoff erosion, increasing water percolation, enhancing soil fertility, providing shade, fencing, and windbreak. Besides, it has a huge potential to adapt to the adverse impact of climate change and mitigate the long-term climate change impact through carbon sequestration^46^. Key informants reported that farmers traditionally used to grow fodder on their land. In the study area, about 70.82% of none-adopters were not using fodder planting. This is mainly due to the problem of free grazing (58.19%), land allocation for crop production (13.36%), small land (7.88%), unavailability of adequate water in all year round a year (12.73%), lack of improved fodder seed (4.24%), and other (0.6%) (see Table 12). Reference^47^ also indicated that land size and availability of information are determining factors for adoption.

Proper pasture management through rotation grazing would be the most cost-effective way to mitigate GHG emissions from feed crop production and through grassland carbon sequestration. Animal grazing on pasture also helps reduce emissions attributable to animal manure storage^19^. Noneadaptor farmers had different constraints as a reason for not practicing rotational grazing. Table 12 shows that 23.76% of the respondents could not adopt rotational grazing practice mainly due to lack of awareness and knowledge its benefits, small grazing land (53.59%), weak land use policy enforcement (11.6%), and communal ownership of the grazing land (11.05%). The finding of^46^ supports this result in that overgrazing and the absence of land use policy in Ethiopia are the major constraints in adopting planned pasture management practices.

Biogas units can be used to convert human and animal waste into a mixture of methane and carbon dioxide that can be used for lighting, heating, and cooking^47,48^. The KIIs reported that Biogas technology also saves women and girls’ labor and hence, invest their time and energy for productive activities, prevents deforestation by substituting firewood, improves garden vegetable production through the usage of by-products from biogas generation as organic fertilizer, and also protects women from the health adverse impact of smoke.

Table 6 shows that only 3.86% of sampled farmers adopted the technology and the remaining 96.14%) did not adopt it due to lack of information and awareness (45.54%), lack of finance (43.75%), a small quantity of manure (8.93%), and other factors (1.79%). This technology is the least adopted (3.86%) practice in the study area. Besides, key informant indicated that shift in household livestock number, management and technical capacity are additional challenges in adopting and maintaining biogas technology. Reference^1^ also indicated that high investment costs are a challenge to adopting climate smart technologies.

Livestock is unable to find adequate fodder and hence, grow weak and die from malnutrition or disease. The availability of supplementary grain and fodder on the local market has been decreasing. As a result, livestock prices drop too low and the price of grain climbs too high. Emergency destocking programs allow for the removal of animals before they die. Destocking livestock is the reduction of the number of livestock, especially during a shortage of feed and water to adjust the number of livestock with their feed capacity. As indicated in Table 12, about 36.5% of the sample farmers were not adopting the destocking of livestock to cope with climate variability induced shortage of water and pasture. The main reasons are low awareness of the destocking (69.05%), cultural attitude (25%), and others (5.95%).

## Conclusions and recommendations

Farmers in Hidabu Abote district adopted various climate smart livestock production practices, including composting, manure management, feed conservation, destocking, improved breeds, fodder planting, rotational grazing, and biogas generation. Among climate smart livestock production practices, composting emerged as the most widely adopted technology, followed by manure management, feed conservation, and destocking, while biogas generation had the lowest adoption rate. The adoption of composting and manure management, alongside limited uptake of biogas generation, suggests that farmers prioritize both environmental sustainability and commercial viability.

The study also identified high cost of improved breeds, limited availability of feed, reliance on manure for fuel and crop residue for animal feeding, issues related to free grazing, low awareness among farmers, small grazing land, and lack of financial resources as barriers to the adoption of climate smart livestock production practices in the study areas. Consequently, farming households with higher levels of education, larger grazing lands, greater livestock holdings, and more frequent contact with extension services were found to be more inclined to adopt climate smart livestock production practices, while others faced challenges in doing so.

To address these barriers, concentrated collaborative efforts between government and non-governmental organizations are recommended. These efforts should focus on enhancing existing initiatives aimed at mitigating identified challenges. Strengthening communication channels with agricultural extension agents, increasing media exposure, and raising awareness about climate change impacts are essential steps in promoting the adoption of climate-smart agricultural practices. Furthermore, there is a necessity to improve the accessibility of feed and advisory support services. Hence, farmers should be encouraged to manage grazing lands effectively, with guidance from agricultural extension experts, to prevent land degradation and overgrazing.

## Data Availability

This article is part of MSc thesis submitted to Addis Ababa University, College of Development Studies, Center for Food Security Studies. The raw data have been submitted to the university registrar. Therefore, anyone who is interested to access the raw data could officially contact Addis Ababa University Library directorate office. However, we could not submit or link the data source due to university regulations.
